# The role of nurses in transforming health care

**DOI:** 10.1186/1472-6963-14-S2-P59

**Published:** 2014-07-07

**Authors:** Carmen Planas-Campmany, M Teresa Icart-lsern

**Affiliations:** 1 Direcció General de Planificació i Recerca en Salut, Barcelona 08028, Spain; 2 School of Nursing. University of Barcelona, Barcelona 08907, Spain

## Background

Health care (HC) planning strategies aim to transform HC models through integrated care schemes and increasing the response capacity of the primary health care (PHC) services. These reforms will require the provision of new roles that are inherently aligned with the nursing model of care [1]. A reflection is needed about the present and future of the Spanish health care system and the role of nurses.

## Materials and methods

The deliberation is based on a textual reading of the Organisation for Economic Co-operation and Development (OECD) statistics of practicing nurses from 2000 to 2011 and the description of the recent situation in the health care area [2].

## Findings

It is widely accepted that nurses play a critical role in providing health care, especially in PHC and in home care settings. The need for health promotion, preventive interventions and caring for people with chronic conditions suggests that nurses are expected to gain importance. Recent data shows an increased number of nurses per capita in almost all OECD countries. Spain showed one of the largest increases since 2000. However, the number of nurses per capita remained well below the OECD average, with a relatively low number of practicing nurses per practicing physicians (1.39) compared to the 2,8 average ratio in OECD countries [2].

## Conclusions

Governments have to become aware of nurses’ contribution. Likewise, nurses have no choice but to position themselves in this new reality, identifying and using their knowledge, their vision of individuals and the ability to provide a genuine perspective based on people’s needs. Furthermore, new questions have to emerge regarding nurses and the evolving health policies, regulation, financing and provision and education [3]. For all of these aspects have a direct impact upon nurses and vice versa.
